# A Novel ‘Gene Insertion/Marker Out’ (GIMO) Method for Transgene Expression and Gene Complementation in Rodent Malaria Parasites

**DOI:** 10.1371/journal.pone.0029289

**Published:** 2011-12-27

**Authors:** Jing-wen Lin, Takeshi Annoura, Mohammed Sajid, Séverine Chevalley-Maurel, Jai Ramesar, Onny Klop, Blandine M. D. Franke-Fayard, Chris J. Janse, Shahid M. Khan

**Affiliations:** Leiden Malaria Research Group, Department of Parasitology, Leiden University Medical Centre, Leiden, The Netherlands; Seattle Biomedical Research Institute, University of Washington, United States of America

## Abstract

Research on the biology of malaria parasites has greatly benefited from the application of reverse genetic technologies, in particular through the analysis of gene deletion mutants and studies on transgenic parasites that express heterologous or mutated proteins. However, transfection in *Plasmodium* is limited by the paucity of drug-selectable markers that hampers subsequent genetic modification of the same mutant. We report the development of a novel ‘gene insertion/marker out’ (GIMO) method for two rodent malaria parasites, which uses negative selection to rapidly generate transgenic mutants ready for subsequent modifications. We have created reference mother lines for both *P. berghei* ANKA and *P. yoelii* 17XNL that serve as recipient parasites for GIMO-transfection. Compared to existing protocols GIMO-transfection greatly simplifies and speeds up the generation of mutants expressing heterologous proteins, free of drug-resistance genes, and requires far fewer laboratory animals. In addition we demonstrate that GIMO-transfection is also a simple and fast method for genetic complementation of mutants with a gene deletion or mutation. The implementation of GIMO-transfection procedures should greatly enhance *Plasmodium* reverse-genetic research.

## Introduction

Reverse genetic technologies have been widely applied to gain an understanding of the function of genes in *Plasmodium* and to provide insight into the biology of malaria parasites and interactions with their hosts (for reviews see [1]–[3]. The availability of efficient genetic modification technologies for the rodent malaria parasites *P. berghei* and *P. yoelii* and the possibilities for analysis of these parasites throughout the complete life cycle have made *P. berghei* and *P. yoelii* the most frequently used models for analysis of gene function [2]. Targeted disruption or mutation of genes coupled with protein tagging has provided insight into *Plasmodium* gene function and parasite protein expression, localization and transport. Reverse genetics is not only applied to understand *Plasmodium* gene function by gene deletion but is also increasingly being used to generate parasites that express heterologous proteins, for example parasites having transgenes introduced into their genome to encode fluorescent or luminescent reporter proteins. Such reporter parasites have been instrumental in the visualization and analysis of parasite-host interactions in real-time *in vitro* and *in vivo* [4]–[6]. The use of mutant parasites to investigate host-parasite interactions as well as parasite gene function requires genetic modification systems that are flexible and easy to perform. The application of reverse genetics in *P. berghei* and *P. yoelii* is however restricted by the limited number of drug resistance genes (permitting the selection of transformed parasites) that are currently available. This low number of selection markers hampers and slows down successive modifications in the genome of the same parasite line. Currently only two resistance gene/drug combinations exist for use in rodent malaria parasites that can be used in successive transfections, specifically *dhfr-ts*/pyrimethamine and h*dhfr*/WR99210 [7]. Since both drug-selection markers confer resistance against pyrimethamine, the introduction of consecutive genetic modifications in the same parasite can only be performed by first selecting with pyrimethamine followed by WR99210 selection [7]. In order to circumvent the problem of limited drug-selection markers, GFP has been utilized as a selection marker and permits the selection of transformed *P. berghei* parasites by flow cytometry [8], [9]. In addition, a method has been developed for removing drug-selection markers from transformed *P. berghei* parasites by utilizing the yeast *fcu* (y*fcu*) selection marker and negative selection with the drug 5-fluorocytosine (5-FC) [10], which kills all parasites expressing *yfcu*. In this method transformed parasites expressing the fusion gene h*dhfr*::y*fcu* are first selected by positive selection with pyrimethamine. Subsequently, negative selection with 5-FC is applied to select for marker-free parasites that have ‘spontaneously’ lost the h*dhfr*::y*fcu* marker from their genome, achieved by a homologous recombination/excision event around the selection cassette [10]. Both the selection of GFP-expressing mutants by flow cytometry and selection of ‘spontaneous’ marker-free mutants by negative selection have their limitations. They are laborious and time consuming, and also require the use of many extra animals as additional cloning steps in mice are required; therefore these methods are not commonly used for successive genetic modifications or for complementation studies [11].

Here we report the development and application of a novel ‘gene insertion/marker out’ (GIMO) system for transfection of two rodent malaria parasites, *P. berghei* and *P. yoelii*. For both species we have created reference mother lines that contain the h*dhfr*::y*fcu* selection marker stably integrated into the silent *230p* genomic locus. We show that transfection of these mother lines with DNA-constructs that target the modified *230p* locus, followed by negative selection of transformed parasites with 5-FC is a simple and fast method to generate mutants that stably express heterologous proteins and are free of drug-selectable markers. These mother lines are therefore useful tools to generate a wide range of mutants expressing reporter and/or other heterologous proteins (under the control of different promoters) without restricting subsequent modification of the genome of these parasites. In addition, we demonstrate that GIMO-transfection is a simple and fast method to genetically complement, restoring the wild-type genotype of parasite mutants with a gene deletion or gene mutation. Importantly, GIMO transfection can be easily partnered for use with a recently developed ‘recombineering’ system for high-throughput, genome wide and highly efficient generation of gene targeting constructs [12].

## Results

### Generation of the *P. berghei* and *P. yoelii* ‘gene insertion/marker out’ (GIMO) mother lines

**Figure 1:**
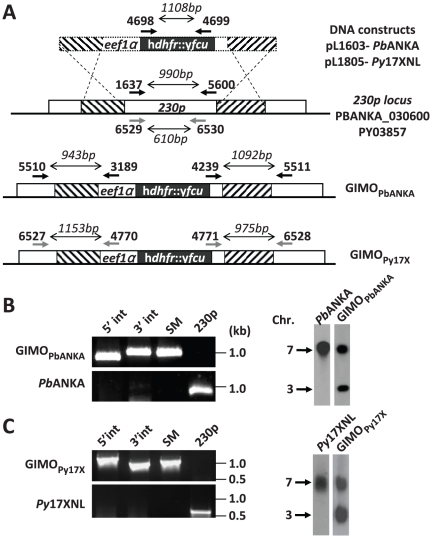
Generation and genotype analyses of *P. berghei* and *P. yoelii* GIMO mother lines. (**A**) Schematic representation of the constructs used to introduce the positive-negative selectable maker cassette in the *P. berghei* (*Pb*ANKA) or *P. yoelii* (*Py*17XNL) *230p* locus. DNA constructs pL1603 (targeting *P. berghei 230p*, PBANKA_030600) and pL1805 (targeting *P. yoelii 230p*, PY03857) containing a fusion of the positive drug selectable marker h*dhfr* (human *dihydrofolate reductase*) and negative marker y*fcu* (yeast *cytosine deaminase* and *uridyl phosphoribosyl transferase*) under the control of the *eef1α* promoter target the *230p* locus at the target regions (hatched boxes) by double cross-over homologous recombination. Location of primers used for PCR analysis and sizes of PCR products are shown (see Table S2 for all primer sequences). (**B**) Diagnostic PCR and Southern analysis of PFG-separated chromosomes confirming correct integration of the construct in the *P. berghei* mother line GIMO_PbANKA_: 5′ integration PCR (5′ int; primers 5510/3189), 3′ integration PCR (3′ int; primers 4239/5511), amplification of h*dhfr*::y*fcu* marker (SM; primers 4698/4699) and the original *P. berghei 230p* (230p; primers 1637/5600). Primer location (black arrows) and product sizes are shown in A. For Southern analysis, PFG-separated chromosome were hybridized using a 3′UTR *pbdhfr* probe that recognizes the construct integrated into *P. berghei 230p* locus on chromosome 3 and the endogenous locus of *dhfr/ts* on chromosome 7. (**C**) Diagnostic PCR and Southern analysis of PFG-separated chromosomes confirming correct integration of the construct in the *P. yoelii* mother line GIMO_Py17X_: 5′ integration PCR (primers 6527/4770), 3′ integration PCR (primers 4771/6528), amplification of h*dhfr*::y*fcu* marker (primers 4698/4699) and the *P. yoelii 230p* original locus (primers 6529/6530). Primer location (grey arrows) and product sizes are shown in A. For Southern analysis, chromosomal hybridization using a 3′UTR *pbdhfr* probe recognizes the construct integrated into *P. yoelii 230p* locus on chromosome 3 and the endogenous locus of *dhfr/ts* on chromosome 7.

For both *P. berghei* ANKA and *P. yoelii* 17XNL transgenic parasites were generated that express a fusion of a drug resistance gene and a drug sensitivity gene, the so called postive-negative selectable marker (SM), constitutively expressed by the *P. berghei eef1α* promoter (Figure 1A). Specifically, these parasites contain a fusion gene of *hdhfr* (human *dihydrofolate reductase*; positive SM) and y*fcu* (yeast *cytosine deaminase* and *uridyl phosphoribosyl transferase*; negative SM) stably integrated into the *230p* locus (PBANKA_030600 in *P. berghei* and PY03857 in *P. yoelii*) through double cross-over recombination. These lines are named GIMO mother lines (gene insersion/marker out); for *P. berghei* GIMO_PbANKA_ (line 1596cl1) and for *P. yoelii* GIMO_Py17X_ (line 1923cl1). Both GIMO mother lines were cloned after transfection by positive selection with pyrimethamine. Correct integration of the h*dhfr*::y*fcu* selectable marker cassette in the *230p* locus was demonstrated by PCR and Southern analysis of chromosomes separated by pulse-field gel electrophoresis (Figure 1B and 1C). The multiplication rate of asexual blood stages per 24 h as determined in mice infected with a single parasite [13], gametocyte production and production of oocysts and sporozoites were identical to those of the parent *P. berghei* and *P. yoelii* lines (data not shown). These GIMO mother lines are used for introduction of transgenes into the modified *230p* locus through transfection with constructs that target the *230p* locus. These constructs insert into the *230p* locus (‘gene insertion’), thereby removing the h*dhfr*::y*fcu* selectable marker (‘marker out’) from the genome of the mother lines. Transgenic parasites that are marker-free are subsequently selected by applying negative drug selection using 5-FC (see below).

### Assessing the efficiency of GIMO-transfection to select transgene expressing, drug-selectable marker-free *P. berghei* parasites

**Figure 2:**
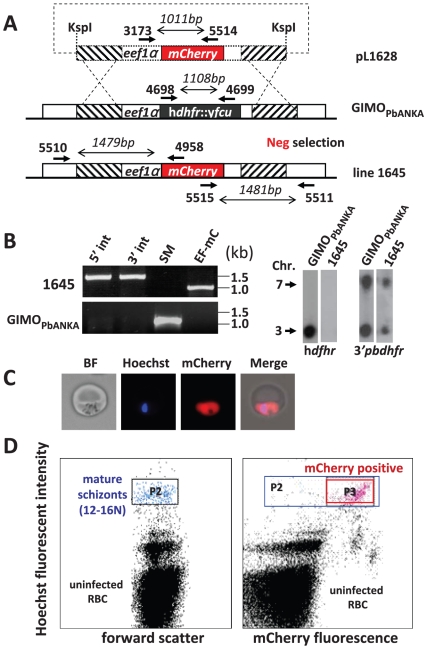
Generation of a marker-free mCherry-expressing parasite using GIMO-transfection. (**A**) Schematic representation of the introduction of a mCherry-expression cassette into the GIMO_PbANKA_ mother line. Construct pL1628 containing the *eef1α*-*mCherry-3′pbdhfr* cassette (mCherry; red box) is integrated into the modified *P. berghei 230p* locus containing the h*dhfr*::y*fcu* selectable marker cassette (black box) by double cross-over homologous recombination at the target regions (hatched boxes). Negative (Neg) selection with 5-FC selects for parasites (line 1645) that have mCherry reporter introduced into the genome and the h*dhfr*::y*fcu* marker removed. Location of primers used for PCR analysis and sizes of PCR products are shown (see Table S2 for primer sequences). (**B**) Diagnostic PCRs and Southern analysis of PFG-separated chromosomes confirms the correct integration of construct pL1628 in line 1645 parasites shown by the absence of the h*dhfr*::y*fcu* marker and the presence of the *mCherry* gene: 5′ integration PCR (5′ int; primers 5510/4958), 3′ integration PCR (3′ int; primers 5515/5511), amplification of h*dhfr*::y*fcu* (SM; primers 4698/4699) and the *eef1α*-mCherry (EF-mC; primers 3173/5514). Primer locations and product sizes are shown in A (primer sequences in Table S2). Hybridization of separated chromosomes of GIMO_PbANKA_ and line 1645 using a h*dhfr* probe recognizes the h*dhfr*::y*fcu* marker in the *230p* locus on chromsomse 3 in GIMO_PbANKA_ but is absent in line 1645. Hybridization with 3′UTR *dhfr* probe recognizes both modified the *230p* locus on chromosome 3 (both marker and *mCherry* expression cassettes contain the 3′*pbdhfr* sequence) and the endogenous *dhfr/ts* gene on chromosome 7 as loading control. (**C**) Fluorescence microscopy of a live mCherry-expressing trophozoite of line 1645; bright field (BF), DNA staining (Hoechst; Blue) and mCherry expression (red). (**D**) FACS analysis of mCherry-expressing blood stages of line 1645. The percentage of mCherry-expressing parasites was performed by FACS analysis on cultured blood stage. Mature schizonts (12–16 N) were selected based on their Hoechst fluorescent intensity (gate P2) and mCherry-expressing schizonts were selected in gate P3 (right panel).

We generated a test DNA-construct containing a transgene expression-cassette to test the efficiency of selection of transgenic mutants through the application of negative selection using 5-FC after transfection into the GIMO_PbANKA_ mother line. This construct contains the *mCherry* gene under the control of the constitutive *eef1α* promoter and *230p* targeting sequences (Figure 2A) and lacks a drug selectable marker cassette. This DNA-construct, pL1628, targets the same regions in the *230p* locus in which h*dhfr*::y*fcu* selection cassette was introduced in the GIMO_PbANKA_ mother line (Figure 2A). Transfection of GIMO_PbANKA_ (exp. 1645) was performed using standard procedures [14] except that after transfection negative drug selection was applied instead of positive drug selection. This negative selection was performed by treating mice that were infected with transfected parasites with the drug 5-FC for 4 consecutive days (one dose per day of 10 mg), starting 24 hours after transfection.

Transfected parasites of line 1645 were collected at day 7 and 8 after transfection (at a parasitemia of 0.5–3%) for phenotype and genotype analyses. Diagnostic PCR and Southern analysis of separated chromosomes confirmed the correct integration of the test construct and simultaneous removal of the h*dhfr*::y*fcu* selection cassette (Figure 2B). Analysis of mCherry expression by fluorescence microscopy in blood stage parasites of line 1645 showed that >90% of the parasites expressed mCherry (Figure 2C). Quantification of the percentage of mCherry-expressing parasites was performed by FACS analysis of mature schizonts collected from overnight blood stage cultures. Expression of transgenes, such as *mCherry*, under the control of the *eef1α* promoter increases with the maturation of parasites inside blood cells and therefore FACS quantification is improved by analysing mature schizont stages (these stages are selected based on Hoechst-fluorescence) [15]. FACS analysis confirmed that >90% (93%±1.1 Figure 2D) of the schizonts were mCherry positive. Since episomal constructs cannot be maintained during selection in GIMO-transfected parasites (see Discussion), these analyses demonstrate that GIMO-transfection permits the selection parasites that express transgenes and are marker-free.

**Figure 3:**
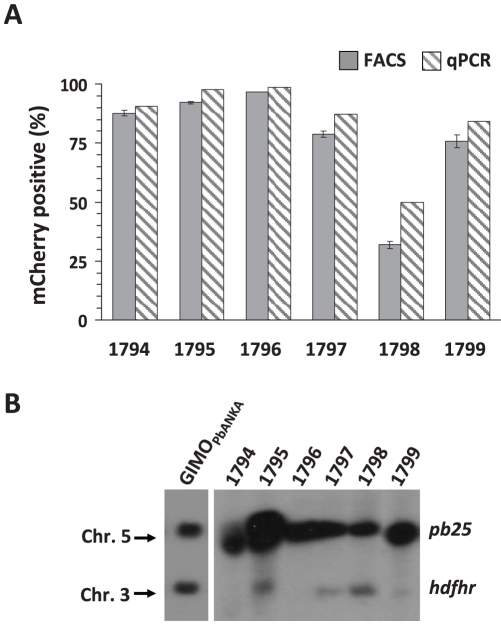
The efficiency of GIMO-transfection to select marker-free parasites that express mCherry. (**A**) Percentage of mCherry-positive parasites in GIMO-transfection of GIMO_PbANKA_ (shown in Figure 2) after negative selection. The percentage of mCherry-positive parasites in six independent transfections (1794–1799) was determined by FACS analysis (see Figure 2D) and quantitative PCR (qPCR). By qPCR the ratio of mCherry and h*dhfr*::y*fcu* marker positive parasites was determined relative to the presence of a control gene *hsp70*, using the 2^−ΔΔCT^ method (primers used in qPCR are described in Table S2). (**B**) Efficiency of selection of h*dhfr*::y*fcu* marker-free determined by Southern analysis of PFG-separated chromosomes. Hybridization performed using a mixture of two probes, one specific for *pb25* (chromosome 5) and one for h*dhfr* (chromosome 3) showing the efficiency of selecting h*dhfr*::y*fcu* marker-free parasites in the different experiments.

To further investigate the efficiency of the GIMO system, we performed a set of independent transfections with the DNA-construct pL1628 (exp. 1794–1799) in the GIMO_PbANKA_ mother line. In these experiments transfected parasites were selected using negative selection as described above and mCherry expression analysed by FACS (Figure 3A). In 5 out of 6 transfection experiments, the percentage of mCherry-expressing parasites was higher than 75%, whereas in one experiment (exp. 1798) 32% of schizonts were mCherry positive (Figure 3A). The presence of mCherry negative parasites in the drug-selected population indicates that non-transformed parasites survived the drug-selection but presumably still carry the h*dhfr*::y*fcu* cassette. We therefore analysed the genotype of the selected populations of all experiments by quantitative real-time PCR (qPCR) and Southern analysis of separated chromosomes to determine the ratio between parasites with and without *hdhfr::yfcu*. For qPCR, C_T_ values of amplification of mCherry, h*dhfr*::y*fcu* and the control *hsp70* gene were determined and the percentage of mCherry positive parasites was calculated as the relative ratio between *mCherry* and h*dhfr*::y*fcu* using the 2^−ΔΔCT^ method [16]. The percentage of mCherry positive parasites based on qPCR correlated well with the percentage determined by FACS analysis (Figure 3A). Southern analysis also showed that in the selected populations a low percentage of parasites still contain the h*dhfr*::y*fcu* gene (Figure 3B). These observations indicate that the application of negative selection after transfection of GIMO_PbANKA_, while it highly enriches for transformed parasites, it does not generate a pure population of marker-free parasites. Therefore, parasite cloning after negative selection is an essential step in GIMO-transfection in order to obtain correctly transformed parasites that express the transgene and are drug-selectable marker free.

### Generation of a *P. yoelii* reporter line, *Py*GFP-luc_con_, which is marker-free and expresses a GFP-luciferase fusion protein, by GIMO-transfection

**Figure 4:**
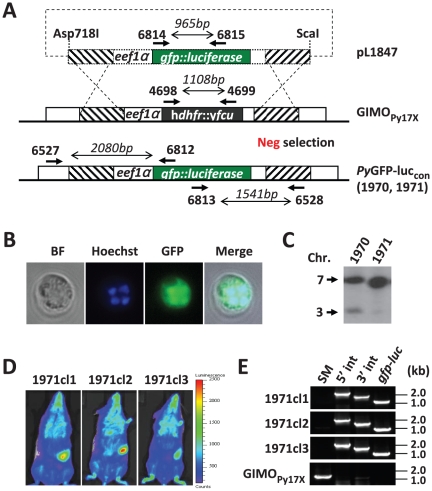
Generation of a *P. yoelii* reporter line, *Py*GFP-luc_con_ that is marker-free and expresses a fusion protein of GFP and luciferase. (**A**) Schematic representation of the introduction of a *gfp-luciferase*-expression cassette into the GIMO_Py17X_ mother line. Construct pL1847 containing the *eef1α*-*gfp::luciferase-3′pbdhfr* cassette is integrated into the modified *P. yoelii 230p* locus containing the h*dhfr*::y*fcu* selectable marker cassette (black box) by double cross-over homologous recombination at the target regions (hatched boxes). Negative selection with 5-FC results in selection of parasites that have the *gfp-luciferase* reporter introduced into the genome and the h*dhfr*::y*fcu* marker removed. Location of primers used for PCR analysis and sizes of PCR products are shown (see Table S2 for primer sequences). (**B**) Fluorescence microscopy of a live schizont of *Py*GFP-luc_con_; bright field (BF), DNA staining (Hoechst; Blue) and GFP expression (green). (**C**) PFG-separated chromosomal Southern analysis of two independent GIMO transfection parasite lines (exp. 1970 and 1971). Hybridization performed with a mixture of two probes, one specific for *pb25* (chromosome 5) and the other for h*dhfr* (chromosome 3), demonstrating the efficiency of selection of h*dhfr*::y*fcu* ‘marker-free’ parasites in the different experiments. (**D**) Analysis of luciferase-expression of blood stages of 3 clones of *Py*GFP-luc_con_ (exp. 1971). Luciferase-activity was measured by real time *in vivo* imaging of live mice with a parasitemia of 1–3%. (**E**) Diagnostic PCR analysis confirming correct integration of the *gfp-luciferase* gene in *Py*GFP-luc_con_ clones (exp. 1971): amplification of h*dhfr*::y*fcu* marker (SM, primers 4698/4699), 5′ integration PCR (5′ int, primers 6527/6812), 3′ integration PCR (3′ int, primers 6813/6528) and *gfp-luc* (primers 6814/6815). Primer location, product sizes are shown in A and primer sequences in Table S2.

The application of negative selection to genetic modification of *P. yoelii* has not been reported. To test the possibility to select *P. yoelii* parasites lacking h*dhfr*::y*fcu* from a population of *hdhfr*::*yfcu*-containing parasites by negative selection, we generated a construct (pL1847) that targets the modified *py230p* locus of the GIMO_Py17X_ mother line by double cross-over homologous recombination. Plasmid pL1847 contains a fusion gene of *gfp* and *luciferase* under the control of the *P. berghei eef1α* promoter (Figure 4A). Integration of this construct will result in the introduction of the *gfp-luc* expression cassette and a simultaneous removal of the h*dhfr*::y*fcu* gene from GIMO_Py17X_ (Figure 4A). Transfection of GIMO_Py17X_ parasites and negative selection was performed as described above for GIMO_PbANKA_. Comparable to results obtained with the transfection of GIMO_PbANKA_, two mice (exp. 1970 & 1971) that were infected with GIMO_Py17X_ transfected parasites became positive at day 6 (parasitemia 1–2%) after selection with the drug 5-FC. Analysis by fluorescence microscopy showed that ∼30% and ∼70% of the parasites of line 1970 and 1971, respectively, were GFP positive (Figure 4B). Southern analysis of PFG-separated chromosomes confirmed that most drug-selected parasites of line 1971 had removed the h*dhfr*::y*fcu* selectable marker (Figure 4C). We obtained three clones of line 1971 and all three expressed luciferase as shown by *in vivo* imaging of mice infected with 1971cl1-3 blood stages parasites (Figure 4D). PCR analysis confirmed the correct integration of the fusion gene *gfp-luciferase* and removal of h*dhfr*::y*fcu* (Figure 4E). The results demonstrate that GIMO-transfection and the negative selection procedure can be applied to *P. yoelii* in order to generate parasites that express transgenes and are free of drug-selectable markers. In addition, these marker-free *P. yoelii* 1971 cloned lines (*Py*GFP-luc_con_), are excellent tools to quantitatively analyse *P. yoelii* development in blood and liver stages using both *in vivo* and *in vitro* luminescent assays as has been achieved with *P. berghei* reporter parasites [17], [18].

### GIMO-transfection is a rapid and simple method for gene complementation

**Figure 5:**
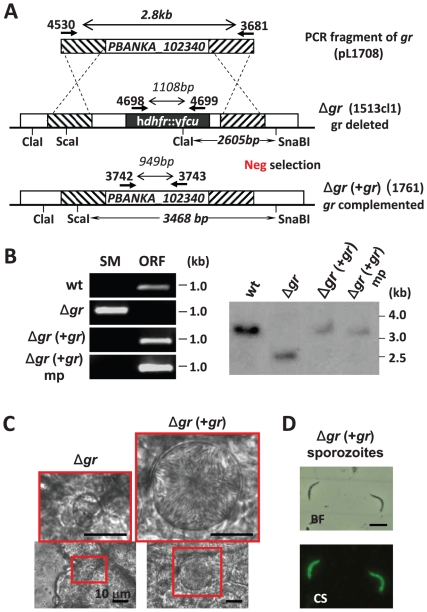
Gene complementation using GIMO-transfection. (**A**) Schematic representation of the re-introduction of the glutathione reductase (*gr*) gene into the *gr* gene deletion mutant (Δ*gr*, 1513cl1); 1513cl1 expresses the h*dhfr*::y*fcu* selectable marker (black box). Transfection with a 2.8 kb PCR-fragment amplified from wild type genomic DNA (primers 4530/3681) containing the *gr* gene, as well as the 5′- and 3′-targeting sequences, was used to re-introduce *gr* gene into the Δ*gr* mutant. Negative selection with 5-FC selects for parasites that have the *gr* gene re-introduced into the genome replacing the h*dhfr*::y*fcu* marker (line 1761; Δ*gr*(+*gr*). Location of primers used for PCR analysis, sizes of PCR products, restriction enzyme sites and sizes of the expected fragments in Southern analysis are indicated (see Tables S1 and S2 for primer sequences). (**B**) Diagnostic PCR analysis and Southern analysis of restricted genomic DNA confirm correct integration of the PCR fragment and complementation in Δ*gr*(+*gr*) parasites: amplification of h*dhfr*::y*fcu* marker (SM; primers 4698/4699) and *gr* (ORF; primers 3742/3743). Primer location, product sizes are shown in A and primer sequences in Table S2. Southern blot was hybridized with 3′UTR *gr* probe (i.e. 3′ targeting region). The localization of the restriction enzymes used and the expected size of the fragments are shown in A: wt (wild type); Δ*gr* (*gr* deletion mutants); Δ*gr*(+*gr*) (complemented Δ*gr*); mp (blood stages after mosquito passage). (**C**) Oocyst development of Δ*gr* and Δ*gr* (+*gr*) parasites. Only small, aberrant oocysts with no signs of sporozoite formation are present in Δ*gr* infected mosquitoes at days 10–21 after feeding. In Δ*gr* (+*gr*) infected mosquitoes sporozoite-containing oocysts with wild-type morphology are visible at day 12. (**E**) Salivary gland sporozoites of Δ*gr*(+*gr*) examined by immuno-fluorescence microscopy: bright field (BF) and anti-CS antibody staining (CS, green).

Gene complementation is used to prove that the phenotype of a gene deletion/modified parasite is the direct result of the gene mutation and not a consequence of an unintended alteration of the parasites genome [11]. Complementation is performed by reintroduction of a wild-type copy of the gene into the genome of a mutant in order to restore the wild-type phenotype, thereby establishing the association of the phenotype to the deletion genotype. We analysed whether GIMO-transfection can be used for gene complementation using a published gene deletion mutant of *P. berghei* with a defined phenotype. Complementation of a mutant using GIMO-transfection requires that the mutant contain the negative selectable marker *yfcu* in its genome. We therefore choose to complement a *P. berghei* mutant (Δ*gr*) which lacks expression of glutathione reductase [19]. In this mutant, the *glutathione reductase* (*gr*) has been deleted using a construct containing the h*dhfr*::y*fcu* marker and the mutant becomes arrested in the mosquito during oocyst development with a complete absence of sporozoite production [19]. For complementation of the Δ*gr* mutant we generated a restoration DNA-construct by simply amplifying the *gr* gene from wild-type *P. berghei* genomic DNA and therefore avoided any cloning steps. Using the same primers that amplified the 5′ and 3′ targeting regions for the DNA construct used to generate the Δ*gr* gene deletion mutant [19] (See Table S1), specifically the forward primer of 5′ targeting region and reverse primer of 3′ targeting region, a 2.8 kb PCR product that contained the complete *gr* gene and both targeting regions was amplified by a high fidelity proof reading polymerase (see Figure 5A). This PCR product was used to transfect Δ*gr* parasites, with the aim to introduce the complete *gr* gene (‘gene insertion’) and thereby replacing the deleted *gr* locus, containing the h*dhfr*::y*fcu* (‘marker out’) as shown in Figure 5A. Selection of transfected parasites, using negative selection was as described above for other GIMO-transfections, and resulted in the selection of parasites (exp. 1761; Δ*gr*(+*gr*)) in which the deleted *gr* had been replaced by the wild-type *gr* gene as confirmed by both diagnostic PCR and Southern analysis of digested genomic DNA (Figure 5B). We next analysed the phenotype of the complemented Δ*gr*(+*gr*) parasites by comparing oocyst and sporozoite development of Δ*gr*(+*gr*) and Δ*gr* parasites in *Anopheles stephensi* mosquitoes. As previously reported [19], Δ*gr* produced oocysts that abort development resulting in small degenerated oocysts without any signs of sporoblast or sporozoite formation (Figure 5C) at day 12 post infection (p.i.). The Δ*gr* infected mosquitoes are not able to infect naive mice at day 21 p.i. In contrast, the complemented Δ*gr*(*+gr*) have normal development in mosquitoes producing normal sized mature oocysts, which contain sporozoites at day 12 p.i. and salivary glands contained sporozoites at day 21 p.i. (Figure 5D). The Δ*gr*(+*gr*) sporozoites are infectious as shown by injection of 10^4^ salivary gland sporozoites in two naïve Swiss mice. Both mice developed a blood stage infection with a prepatency period of 5 days which is comparable to the prepatancy of mice infected with 10^4^ wild type sporozoites. Genotype analysis of Δ*gr*(+*gr*) blood stage parasites after mosquito passage and sporozoite infection, by diagnostic PCR and Southern analysis of digested genomic DNA, confirmed that *gr* was indeed restored (i.e. complemented) in the Δ*gr*(+*gr*) parasites and no deletion mutants were present (Figure 5B). The restoration of the phenotype of Δ*gr* parasites using a PCR-amplified construct in combination with negative selection demonstrates that GIMO transfection is a fast method for gene complementation (see also the Discussion section). In addition it is a relatively simple method, requiring only PCR-amplified DNA-constructs that can be used as the constructs do not require a drug-selectable marker cassette.

## Discussion

Genetic modification of malaria parasites is limited by the paucity of drug-selection markers that permit selection of transformed mutants, which in turn hampers the generation of multiple genetic modifications in the same mutant. The novel GIMO-transfection method reported in this study permits the generation of mutants stably expressing heterologous proteins free of drug-selectable markers, facilitating further genetic modification of the transgenic parasites. In addition, it provides a fast and simple way for gene complementation of gene deletion/mutation mutants. We have generated reference mother lines and standard ‘knock-in’ constructs for both *P. berghei* ANKA and *P. yoelii* 17XNL, which we will make available for the research community. In GIMO-transfection of these mother lines, transgenes are introduced in the *230p* locus of both *P. berghei* and *P. yoelii*. For *P. berghei* ANKA it has been shown that *230p* is a ‘silent’ locus [20] and different reporter lines with transgenes introduced in this locus has been generated that show wild-type progression through the complete life-cycle [8], [21]. Whether *230p* is also a ‘silent’ locus in *P. yoelii* has not been reported before. Our observations of normal development of asexual stages, mosquito development and sporozoite infectivity of the *P. yoelii* mother line and *Py*GFP-luc_con_ indicates that *p230* is also a suitable locus to introduce transgenes in *P. yoelii*.

Several *P. berghei* reference lines exist that express reporter proteins, such as GFP and luciferase, and do not contain drug-selection markers. Most of these parasites have been obtained by FACS-sorting where GFP expression is used as the selectable marker [8], [9]. However, selection of transgenic fluorescent-expressing parasites by FACS-sorting has been only reported for selecting GFP-expressing parasites and not with parasites that express other fluorescent proteins. In our hands, FACS-sorting of GFP-expressing parasites is not a highly efficient selection method as often the selected population consists of both mutant and wild type parasites. Moreover, introducing a GFP-selection cassette increases the size of the transfection construct. This limits the size of the heterologous DNA that can be cloned into these vectors as it is difficult to maintain *Plasmodium* transfection vectors with a size larger than 14 kb in *E. coli*. Therefore, in comparison with FACS-sorting, the GIMO-transfection system is a more flexible and simpler system to introduce a wide range of heterologous genes into the parasite genome with the additional advantage that GIMO transfection constructs are far smaller since a selection-marker cassette is not required.

**Figure 6:**
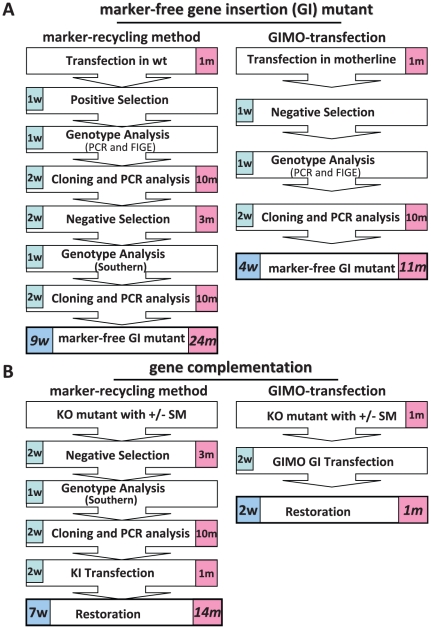
Compared to the marker-recycling method GIMO-transfection is faster and requires fewer animals to both generate marker-free gene insertion (GI) mutants and to complement gene deletion mutants. (**A**) Number of weeks (w) and number of mice (m) needed to generate ‘marker-free’ gene insertion mutants expressing transgenes using GIMO-transfection (right) and using the marker-recycling method (left). (**B**) Number of weeks (w) and number of mice (m) needed for complementation of a gene deletion mutant using GIMO-transfection (right) and using the marker-recycling method (left).

In addition to the use of FACS-sorting for the generation of marker-free *P. berghei* mutants a ‘marker-recycling’ method has also been employed in *P. berghei* [10]. Specifically, transformed parasites expressing the fusion gene h*dhfr*::y*fcu* are first selected by positive selection with pyrimethamine; subsequently negative selection with 5-FC is applied to select parasites that have lost the resistance genes. The efficiency of selection of marker-free parasites is dependent on the frequency of the loss of the h*dhfr*::y*fcu* marker from the genome by homologous recombination and excision [10]. This method has been successfully used to generate marker-free reporter lines [22], to introduce two independent genetic modifications in the same parasite lines [22]–[24] and for complementation [10]. However, this marker-recycling method is relatively laborious and time consuming since it involves both positive and negative selection procedures and two parasite-cloning steps, a procedure requiring at least 9 weeks to complete. Further, marker-recycling method requires at least 24 mice in order to obtain a marker-free mutant (Figure 6A), in part a consequence of essential cloning procedures [19], [22]. In contrast, the generation of marker-free mutants with GIMO-transfection can be achieved in only 4 weeks and requires only 11 mice (Figure 6A). The marker-recycling transfection constructs consist of the h*dhfr*::y*fcu* drug-selectable marker cassette, a transgene expression and two targeting sequences for integration into the genome (See Figure S2A). In addition, they have two identical regions of DNA sequence that can recombine (in the parasite genome) and excise the selectable marker cassette. In contrast the GIMO-constructs contain only the two genome targeting sequences and the transgene expression cassette (see Figure S2 for a comparison of the marker-recycling and GIMO constructs). The simple structure of GIMO constructs permits the cloning of larger transgenes (the GIMO constructs are smaller as the selectable marker cassette is absent) and improves the retention of plasmids in bacteria as internally repetitive regions of AT-rich *Plasmodium* DNA are absent. Further, after transfection with the GIMO construct, the selection of integration mutants is improved as no episomal construct DNA is maintained in the parasites and negative selection kills parasites expressing *yfcu*.

GIMO-transfection is dependent on the transgene-expression construct replacing the h*dhfr*::y*fcu* selection cassette present in the mother line genome and the efficiency of the drug 5-FC to kill all parasites where this integration has not occurred and that are still expressing yFCU. Interestingly, in both *P. berghei* and *P. yoelii* GIMO-transfection experiments we always observed that populations of 5-FC selected parasites contain (low numbers of) parasites that still have the h*dhfr*::y*fcu* selection cassette in their genome. Further research is required to determine whether these parasites express yFCU but are able to survive 5-FC drug treatment or if these parasites have lost expression of yFCU through the mutation of h*dhfr*::y*fcu* selectable marker cassette. Experiments in our laboratory are now focused on improving the application of negative selection to mutant parasites in mice by providing 5-FC in the drinking water, which may permit treatment with higher concentrations of 5-FC and for longer periods. Notwithstanding the presence of non-transformed parasites after selection of GIMO-transfected parasites, the high percentages of transformed parasites in the populations permit the collection of the desired mutants by cloning. Using GIMO- transfection we have already been able to successfully generate multiple marker-free lines that express a variety of heterologous proteins (unpublished data JWL and SK).

GIMO-transfection was used to generate a *P. yoelii* GFP-luciferase reporter parasite and is the first report describing the use of negative selection with 5-FC in combination with the yFCU marker for genetic modification of this parasite species. Moreover, the *Py*GFP-luc_con_ line is the first *P. yoelii* reporter line that is marker-free and can be easily further genetically modified. Similar *P. berghei* reporter lines have been used to visualize and quantify host parasite interactions *in vivo* [13], [21], [25], [26], analysis of drug-susceptibility [17], [27], [28] and *in vivo* quantification of liver stage development [18], [29].

In this study we demonstrate that GIMO-transfection can not only be used to introduce heterologous genes but also is a fast and simple method for gene complementation. Restoration of the wild type phenotype by gene complementation is the most optimal strategy to show that a mutant phenotype is the result of the intended deletion (or mutation) and is not due to unrelated alterations in the parasite genome [2], [11]. Genetic complementation has not been widely applied in *Plasmodium* due to difficulties in making successive genetic modifications in the same parasite, and to problems inherent in cloning full-length AT-rich *Plasmodium* genes into bacterial plasmid vectors [11]. Till now two methods have been used to complement gene deletion mutants in *P. berghei*. The first method re-introduces the wild-type gene using a construct containing *hdhfr* as a positive selectable marker [30], [31]. The encoded protein confers resistance to WR99210, and can be used to transfect gene deletion mutants that already contain the pyrimethamine resistance markers *dhfr/ts* from *P. berghei* or the *dhfr* from *Toxoplasma gondii* (*tgdhfr*) [7]. However, selection with WR99210 is not straightforward because of problems with dissolving this drug and because there is a reduced sensitivity to WR99210 of parasites that already contain the *dhfr/ts* or *tgdhfr* marker [7], [10] (unpublished observations CJJ). The second complementation method is based on the marker-recycling, as described above. Gene deletion mutants (containing *hdhfr*::y*fcu*) are first subjected to negative selection to select for marker-free parasites, cloned and then transfection is performed with constructs containing the gene for complementation and a drug selection cassette [10] (see Figure S3A). This method requires generally 7 weeks and 14 mice to perform (Figure 6B). In contrast, complementation with GIMO-transfection takes only 2 weeks and 1 mouse (Figure 6B). Not only is the GIMO method much faster, requiring far fewer mice, but also a big advantage is that a simple PCR amplicon containing the wild-type gene can be used for complementation as no drug selectable needs to be used in the construct (see Figure S3 for schematics of the marker-recycling and GIMO methods).

In summary, we have developed a novel method that simplifies and speeds up both the generation of marker-free parasites expressing heterologous proteins and for the genetic complementation of gene deletion/mutation mutants. Moreover the application of this method greatly reduces the numbers of animals required to generate and complement mutants. We have also generated the first marker-free *P. yoelii* reporter line and established the successful use of negative selection in transfection of *P. yoelii* parasites. The GIMO-transfection is a simple, fast and efficient approach to generate mutants permissive to subsequent genetic modification. Therefore we recommend that, where possible, transfection of *P. berghei* and *P. yoelii* parasites be performed with constructs that contain the postive-negative selectable marker cassette, h*dhfr*::y*fcu*. The presence of this marker in mutants permits subsequent GIMO transfection that not only simplifies the creation of additional deletions or modifications but also gene complementation experiments. A recent study has reported high-throughput, genome wide and highly efficient ‘recombineering’ system, for high-throughput, genome wide and highly efficient generation of gene targeting constructs [12]. This exciting development can be partnered with GIMO transfection by ensuring all these targeting constructs have a positive-negative (h*dhfr::yfcu*) selectable marker cassette. Consequently all resulting mutants would be receptive to GIMO transfection thereby permitting further modification (e.g. reporter protein expression) and complementation.

## Materials and Methods

### Experimental animals and parasites

Female Swiss OF1 mice (6–8 weeks old; Charles River/Janvier) were used.

All animal experiments of this study were approved by the Animal Experiments Committee of the Leiden University Medical Center (DEC 07171; DEC 10099). The Dutch Experiments on Animal Act is established under European guidelines (EU directive no. 86/609/EEC regarding the Protection of Animals used for Experimental and Other Scientific Purposes).

Two reference rodent malaria parasite lines were used: *P. berghei* ANKA line cl15cy1 [14] and *P. yoelii* 17XNL (clone 1.1) parasite line [32].

### Generation of GIMO mother lines in *P. berghei* ANKA and *P. yoelii* 17XNL

To generate the GIMO mother line in *P. berghei*, a DNA-construct pL1603 was generated for integration into the *230p* gene (PBANKA_030600) by cloning the 5′ and 3′ regions of *230p* as previously described [8]. The targeting sequences were amplified from genomic DNA using primer sets 5585/5586 and 5587/5588 (See Table S1 for the sequence of all primers) and cloned into the restriction sites of HindIII/KspI and Asp718I/EcoRI of the standard cloning vector pL0034 (MRA-849, www.mr4.org), which contains the h*dhfr*::y*fcu* selectable marker under the control of the *eef1α* promoter [10]. The h*dhfr*::y*fcu* marker is a fusion gene of the positive selection marker human *dihydrofolate reductase* and the negative selection marker which is a fusion gene of yeast *cytosine deaminase* and *uridyl phosphoribosyl transferase* [10]. Prior to transfection the DNA-construct pL1603 was linearized with HindIII and EcoRI.

To generate the GIMO mother line in *P. yoelii*, a modified two step PCR method [33] was used to generate DNA-construct pL1805 for integration into the *230p* gene (PY03857) of *P. yoelii* (Figure S1A). In the first PCR reaction two fragments (5′- and 3′- targeting sequences, both ∼1 kb) of *230p* were amplified from *P. yoelii* 17XNL genomic DNA with the primer sets 6523/6524 and 6525/6526 (Table S1). Primers 6524 and 6525 have 5′- extensions homologues to the h*dhfr*::y*fcu* selectable marker cassette (CATCTACAAGCATCGTCGACCTC in 6524 and CCTTCAAfTTTCGGATCCACTAG in 6525). This selectable marker cassette was excised by digestion with XhoI and NotI from a plasmid (pL0048) that contains the *P. berghei eef1α*-h*dfhr*::y*fcu-3′dhfr/ts* (i.e. promoter-drug selectable marker-3′ terminator sequence) selection cassette. Primers 6523 and 6526 have 5′-terminal extensions with an anchor-tag suitable for the second PCR reaction. In the second PCR reaction, the amplified 5′- and 3′- targeting sequences were annealed to either side of the selectable marker cassette, and the joint fragment was amplified by the external anchor-tag primers 4661/4662, resulting in the PCR-based targeting construct with an expected size of 4.7 kb (2.7 kb of the selectable marker cassette plus two targeting sequences of 1 kb). Before transfection, the PCR-based construct was digested with Asp718I and ScaI (in primers 6523 and 6526, respectively) to remove the ‘anchor-tag’ and with DpnI that digests any residual pL0048 plasmid.

Transfection in *P. berghei* ANKA and *P. yoelii* 17XNL, selection and cloning of the mother lines were performed by standard procedures described for transfection of *P. berghei* [14]. DNA-construct pL1603 was introduced into *P. berghei* generating mother line, GIMO_PbANKA_ (1596cl1), and DNA construct pL1805 was introduced into *P. yoelii* generating mother line, GIMO_Py17X_ (1923cl1). Correct integration of the constructs was verified by diagnostic PCR analysis (see Table S2 for primers used) and Southern blot analysis of pulse-field gel (PFG) electrophoresis-separated chromosomes probed with the 3′ untranslated region (UTR) of the *dhfr/ts* gene of *P. berghei*.

### Generation of basic constructs without selection marker and that target the *230p* locus of the GIMO_PbANKA_ and GIMO_Py17X_ mother lines

To generate a basic *P. berghei 230p*-targeting construct (pL0043), the *230p* targeting regions as well as the ampicillin resistance gene were amplified from plasmid pL1063 (MRA-852, www.mr4.org) using primers 5116/5117 (Table S1). A multiple cloning site (MCS) was amplified from pCRII-Blunt-TOPO vector (Zero Blunt TOPO PCR Cloning Kit, Invitrogen, Groningen, The Netherlands) using M13 forward and reverse primers. The two PCR products were digested with *Asp718*I and *Not*I restriction enzymes and ligated together creating the targeting construct pL0043.

A basic *P. yoelii 230p*-targeting construct (pL1849) was generated using a modified 2-step PCR method (Figure S1B). In the first PCR reaction, 5′-and 3′- targeting sequences (both ∼1 kb) of *230p* were amplified from *P. yoelii* 17XNL genomic DNA with the primer set 6523/6534 and 6525/6526 (Table S1). As described above these primers contain 5′- extensions homologues to the h*dhfr*::y*fcu* selectable marker cassette and 5′-terminal extensions with an anchor-tag suitable for the second PCR reaction. A 55 nt oligo (oligo 6598; GAGGTCGACGATGCTTGTAGATGCCCGGGCCTTCAATTTCGGATCCACTAG) containing a XmaI restriction site flanked by 2 sequences homologues to the h*dhfr*::y*fcu* selectable marker cassette was used to join the two *230p* targeting regions (Figure S1B). In the second PCR reaction an fragment containing both *230p* targeting sequences interrupted by the XmaI site was amplified, using the external anchor-tag primers 4661/4662, resulting in the PCR product of ∼2 kb. The PCR product was cloned into TOPO TA vector (TOPO TA Cloning® Kit, Invitrogen, Groningen, The Netherlands) resulting in construct pL1849.

### Generation of a mCherry reporter test construct and GIMO-transfection in the *P. berghei* mother line, GIMO_PbANKA_

A test construct (pL1628) for GIMO-transfection in the GIMO_PbANKA_ mother line was generated by transferring the mCherry-expression cassette (*5′pbeef1α-mCherry-3′pbdhfr*) from plasmid pL0017-mCherry [34] into the basic *230p* targeting construct pL0043 (see above) using restriction sites EcoRV/Asp718I. This plasmid was linearized with KspI before transfection. Transfection was performed as described [14]. Transformed parasites were selected by negative selection by the administration the drug 5-FC (Sigma) to mice infected with transfected parasites. Specifically; 0.4 g/kg bodyweight of 5-FC (stock: 20 mg/ml in 1×PBS) administered by intra-peritoneal injection; one dose per day; for a period of 4 days, starting at 24 hours after transfection. Transformed parasites were collected at day 6/7 (infected tail blood) for phenotype analysis by fluorescence microscopy and FACS (see below) and at day 7/8 (infected heart blood) for genotype analysis using standard methods of diagnostic PCR and Southern analysis of PFG-separated chromosomes [14].

### Generation of a constitutively GFP-luciferase expressing *P. yoelii* (*Py*GFP-luc_con_) reporter line using GIMO-transfection

A construct (pL1847) for GIMO-transfection in the GIMO_Py17X_ mother line was generated by cloning an PCR-amplified GFP-luciferase expression cassette into the XmaI site of the basic *P. yoelii 230p* targeting construct pL1849 (see above). The GFP-luciferase expression cassette (5*′ eef1α-gfp::luciferase-3′pbdhfr*) was amplified from pL1603 (MRA-852, www.mr4.org) using primers 6599 and 6600.

Transfection of GIMO_Py17X_ parasites and negative selection of transformed parasites was performed as described above for transfection of GIMO_PbANKA_. Transformed parasites were collected for genotype analyses using standard methods of diagnostic PCR and Southern analysis of PFG-separated chromosomes [14]. Cloned parasites were analysed for luciferase expression using the *in vivo* imaging technology described below.

### Gene complementation using GIMO transfection

Gene complementation was performed using the published g*lutathione reductase* deletion mutant (Δ*gr*) of *P. berghei* [19]. In this mutant (Δ*gr4*; 1531cl1) the g*lutathione reductase* (*gr*) gene has been deleted by a replacement construct (pL1538) that contains the postive-negative h*dhfr*::y*fcu* selectable marker cassette [19]. The pL1538 construct contains 5′ and 3′ targeting regions of *gr*. We used two of the primers that have been used to generate the replacement construct pL1538 to amplify *gr* gene from *P. berghei* genomic DNA using a proof reading polymerase (Phusion®, Finnzymes, Espoo, Finland). These primers (4049; forward primer for 5′ targeting region and 3681; reverse primer for 3′ targeting region) amplify the complete *gr* gene including the 5′ and 3′ targeting regions (see Table S1 for primer sequences). PCR resulted in amplification of a 2.8 kb fragment which was used to transfect Δ*gr* parasites using standard transfection procedures [14]. Transformed parasites were selected by negative selection as describe above. Transformed parasites were collected for genotype analyses using standard methods of diagnostic PCR and Southern analysis of digested genomic DNA. Analysis of the phenotype of the complemented parasites, Δ*gr*(+*gr*), was analysed by mosquito transmission experiments (see below).

### Fluorescence microscopy and FACS analysis

For analysis of GFP- or mCherry- expression in blood stages of transgenic parasites, infected tail blood was collected in PBS and examined by microscopy using a Leica DMR fluorescent microscope with standard GFP and Texas Red filters. Parasites nuclei were labeled by staining with Hoechst-33258 (2 µmol/L, Sigma, NL). Images were recorded with the digital camera CoolSNAP HQ^2^ (Photometrics, NL) and processed with the ColourProc software [35]. The percentage of blood stages parasites that express mCherry was determined by FACS analysis of cultured blood stages. In brief, infected tail blood (10 µL) with a parastemia between 0.5 and 1% was cultured overnight in 1 mL complete RPMI1640 culture medium at 37°C under standard conditions for the culture of *P. berghei* blood stages [36]. Cultured blood samples were then collected and stained with Hoechst-33258 (2 µmol/L, Sigma, NL) for 1 hr at 37°C in the dark and analysed using a FACScan (BD LSR II, Becton Dickinson, CA, USA) with filter 440/40 for Hoechst signals and filter 610/20 for mCherry fluorescence. For FACS analysis the population of mature schizonts were selected based on the their Hoechst-fluorescence intensity [37]; see gate P2 in the left panel of Figure 2D. The percentage of mCherry-expressing parasites was calculated by dividing the number of mCherry-positive schizonts (gate P3 in right panel of Figure 2D) by the total number of schizonts (gate P2).

### Quantitative real-time PCR (qPCR) analysis of transformed parasites

Genomic DNA extracted from blood stage parasites was used for qPCR analysis. To determine the ratio of transformed/non-transformed parasites in the selected parasite populations, PCR amplifications of the *mCherry* gene (only present in transformed parasites) and the h*dhfr::*y*fcu* selectable marker (only present in non-transformed) were carried out using the QuantiTect SYBR Green PCR Kit (Qiagen, Hilden, Germany) on a CFX96 thermal cycler (Bio-Rad Laboratories, The Netherlands). The housekeeping gene, *P. berghei hsp70*, was used as reference (see Table S2 for primers used). Real-time PCR cycle thresholds (C_T_) were calculated as the average of triplicate analyses (per genomic DNA from transgenic parasite). The ratio between *mCherry* and h*dhfr*::y*fcu* was calculated by the 2^−ΔΔCT^ method relative to *hsp70* [16]. The amplification efficiencies of *mCherry* and *hsp70* did not violate assumptions of the ΔΔC_T_ method (data not shown).

### Real time in vivo imaging of the PyGFP-luc_con_ reporter parasites in whole bodies of live mice

Expression of luciferase and imaging of distribution of luciferase-expressing *Py*GFP-luc_con_ parasites in whole bodies of live mice was determined by measuring bioluminescent activity using the IVIS100 *in vivo* imaging system (Caliper Life Sciences, USA) as described previously [21], [38]. Bioluminescence of blood stage parasites was imaged in Swiss mice with asynchronous infections of *Py*GFP-luc_con_ parasites at a parasitemia of 0.5–2%.

### Analysis of the phenotype of Δ*gr* and complemented Δ*gr*(+*gr*) parasites during mosquito transmission

Infection of *Anopheles stephensi* mosquitoes with *Δgr and Δgr*(*+gr*) parasites as well as determination of production of oocysts and salivary gland sporozoites was performed as previously described [39]. Infectivity of sporozoites was tested by intravenous injection of Swiss OF1 mice with 10^4^ hand dissected salivary gland sporozoites. The prepatent period was determined by light microscopy analysis of Giemsa-stained thin smears of tail blood. Prepatency (measured in days after sporozoite inoculation) is defined as the day when parasitemia reaches 0.5–2%.

### Indirect Immunofluorescence assay

10^4^ *Δgr*(*+gr*) salivary gland sporozoites in 10 µL were allowed to adhere to polylysine coating slides, fixed for 15 minutes with 4% PFA, and washed 3×5 minutes with PBS. Sporozoites were then permeabilized with 0.5% Triton-X100 for 15 minutes followed by a 3×5 minutes wash with PBS. Slides were blocked 30 minutes at room temperature in 10% FCS and incubated over night with polyclonal rabbit anti-CS antiserum [40] (dilution 1∶1000, kindly provided by Dr M. Yuda) at 4°C. Slides were washed 3×5 minutes in PBS and incubated with donkey anti-rabbit, Alexa 488-conjugated secondary antibody (dilution 1∶500), 1 hr in room temperature. Slides were washed 3×5 minutes in PBS, and then incubated 15 minutes with Hoechst 33342 in room temperature. Prior to mounting, slides were washed for 5 minutes and analysed with were analyzed using a Leica DMR fluorescence microscope at 1000× magnification.

## Supporting Information

Figure S1 **Generation of** ***P. yoelii 230p*** **targeting constructs using a PCR method.** **A.** The DNA construct (pL1805) used to generate the *P. yoelii* GIMO mother line was created using a modified two-step PCR method. In the first PCR reaction, 5′- and 3′- targeting sequences of *230p* were amplified from *P. yoelii* 17XNL genomic DNA with the primer sets 6523/6524 and 6525/6526 (Table S1). Primers 6524 and 6525 have 5′- extensions homologues to the h*dhfr*::y*fcu* selectable marker cassette (hatched boxes). This selectable marker cassette was excised from plasmid pL0048 digested with XhoI and NotI. Primers 6523 and 6526 have 5′-terminal extensions (black boxes) for the second PCR reaction. In the second PCR reaction, the 5′- and 3′- targeting sequences annealed to either side of the selectable marker cassette, and the joint fragment was amplified by the external anchor-tag primers 4661/4662. Before transfection, the PCR construct was digested with Asp718I and ScaI to remove the anchor-tag and with DpnI to digest any residual pL0048 plasmid. **B.** The basic *P. yoelii 230p* targeting construct (pL1849) was generated by a modified PCR method. In the first PCR reaction, 5′-and 3′- targeting sequences with homologous sequences (hatched boxes) and anchor-tag sequences (black boxes) were amplified as shown in **A.** Oligo no. 6598 that contains the joint homologous sequences interrupted by an XmaI site (hatched boxes) was used as template for the second PCR reaction. Using the external anchor-tag primers 4661/4662, a PCR product containing both targeting sequences now with the XmaI site in the middle was amplified and subsequently cloned into TOPO TA vector resulting in construct pL1849.
(EPS)

Figure S2 **Schematic representation of the generation of marker-free gene insertion (GI) mutants using GIMO-transfection method or using the marker-recycling method.** **A.** Generation of marker-free gene insertion mutants expressing a gene of interest (GOI; grey box) using the standard marker-recycling method. The construct containing the h*dhfr*::y*fcu* selectable maker (black box) flanked by the recombination sequences (rc, shaded boxes) targets the *230p* locus by double cross-over homologous recombination at specific target regions (hatched boxes). GI mutants are obtained after transfection, using positive selection with pyrimethamine and then cloning. Subsequently, marker-free GI mutants are selected by negative selection using 5-FC. Only those mutants that have ‘spontaneously’ lost the h*dhfr*::y*fcu* marker from their genome, achieved by a homologous recombination/excision (see arrow), survive negative selection. **B.** Generation of marker-free gene mutants that express a GOI (grey box) using GIMO-transfection. The construct that contains no selectable marker cassette and targets the modified GIMO mother line *230p* locus that contains the h*dhfr*::y*fcu* (black box) marker, by double cross-over homologous recombination at the target regions (hatched boxes). Marker-free GI mutants, that have GOI expression cassette introduced into the *230p* locus replacing the h*dhfr*::y*fcu* marker, are obtained by negative selection with 5-FC.
(EPS)

Figure S3 **Schematic representation of gene complementation using GIMO-transfection and the marker-recycling method.** **A.** Gene deletion and complementation using the marker-recycling method. The gene deletion construct, containing the h*dhfr*::y*fcu* selectable maker (black box) flanked by the recombination sequences (rc; shaded boxes), targets the gene of interest (GOI) by double cross-over homologous recombination at the target regions (hatched boxes). Gene deletion mutants are obtained after transfection and positive selection with pyrimethamine, and cloning. Subsequently, marker-free gene deletion mutants are selected by negative selection using 5-FC. Only those mutants that have ‘spontaneously’ lost the h*dhfr*::y*fcu* marker from their genome, achieved by a homologous recombination/excision event (see arrow), survive negative selection.Complementation of the (cloned) marker-free gene deletion mutant is performed using constructs that contain a GOI expression cassette and a positive selectable marker cassette. These constructs can target either the original deleted locus or a locus that is redundant or functionally silent. Complemented parasites are selected by positive selection. **B.** Gene deletion and complementation using the GIMO-transfection method. The gene deletion construct containing the h*dhfr*::y*fcu* selectable maker fusion (black box) targets the GOI by double cross-over homologous recombination at specific target regions (hatched boxes). Gene deletion mutants are obtained after transfection using positive selection with pyrimethamine and then cloning. These constructs do not include recombination (rc) sequences (see A). Complementation of the gene deletion mutant is performed using a PCR fragment amplified from genomic DNA using the same outer primers used to generate the gene deletion construct (i.e. the forward primer of the 5′UTR and the reverse primer of 3′UTR, indicated by arrows). Integration of the PCR fragment by homologous recombination restores the deleted gene locus replacing the h*dhfr*::y*fcu* maker. Complemented parasites are selected by negative selection.
(EPS)

Table S1 **Primers used for DNA construct generation.**
(DOC)

Table S2 **Primers used for genotype analysis.**
(DOC)
