# Supplementary material for: A Novel ‘Gene Insertion/Marker Out’ (GIMO) Method for Transgene Expression and Gene Complementation in Rodent Malaria Parasites
Source: PLoS One. 2011 Dec 27;6(12):e29289. doi: 10.1371/journal.pone.0029289 (PMC3246482; doi:10.1371/journal.pone.0029289)
Supplement: Table S1 — Primers used for DNA construct generation. (DOC) [file pone.0029289.s004.doc]

**Table S1: Primers used for DNA construct generation**

| **DNA**  **Construct** | **No.** | **Primer sequences** | **Restriction**  **sites** | **Descirption** |
| --- | --- | --- | --- | --- |
| pL1603 | 5585 | CTTGGTGACGAAGCTTGTATATGGTAAAGAACCTACTAACAC | *Hind*III | *pb230p* 5’- targeting sequence, F |
|  | 5586 | CTTGGTGACGCCGCGGAGGATGTGTTTTATTTGGATGTG | *Ksp*I | *pb230p* 5’- targeting sequence, R |
|  | 5587 | CCGGGGTACCAATTCTCTTGAGCCCGTTAATG | *Asp718*I | *pb230p* 3’- targeting sequence, F |
|  | 5588 | CCGGGAATTCGTATGGAACTACATCTATATAGG | *EcoR*I | *pb230p* 3’- targeting sequence, R |
| pL1805 | 6523 | GAACTCGTACTCCTTGGTGACGGGTACCGTGATGGAATGGCAACATCTG | *Asp718*I | *py230p* 5’- targeting sequence, F |
|  | 6524 | ***CATCTACAAGCATCGTCGACCTC***GGTTGGACAATGTAATGCTAC |  | *py230p* 5’- targeting sequence, R |
|  | 6525 | ***CCTTCAATTTCGGATCCACTAG***AAGTAAAAGGGGTAAGACAGC |  | *py230p* 3’- targeting sequence, F |
|  | 6526 | AGGTTGGTCATTGACACTCAGCAGTACTAAGAGATCTGGAACCAACTGG | *Sca*I | *py230p* 3’- targeting sequence, R |
|  | 4661 | GAACTCGTACTCCTTGGTGACG |  | anchor-tag primer, F |
|  | 4662 | AGGTTGGTCATTGACACTCAGC |  | anchor-tag primer, R |
| pL0043 | 5116 | GGGGTACCGAGCTCGAATTCTCTTGAGC | *Asp718*I | *pb230p* targeting sequences and *Amp* marker of pL1063, F |
|  | 5117 | ATAGTTTAGCGGCCGCCCTGCAGGCATGCAAGCTTG | *Not*I | *pb230p* targeting sequences and *Amp* marker of pL1063, R |
| pL1847 | 6598 | GAGGTCGACGATGCTTGTAGATGCCCGGGCCTTCAATTTCGGATCCACTAG | *Xma*I | anchor-tag cloning sequence |
|  | 6599 | TCCCCCCGGGGCCCAGCTTAATTCTTTTCGAGCTC | *Xma*I | 5’*pbeef1α*, F |
|  | 6600 | TCCCCCCGGGTTGAAGGAAAAAACATCATTTGTG | *Xma*I | 3’*pbdfhr/ts*, R |
| pL1538 | 4049^*^ | TCCCCGCGGCGATTAAATCATAAACCATATTTG | *Ksp*I | *gr* 5’targeting sequence, F |
|  | 4050^**^ | GCCAAGCTTCAATTACCCAAATTAAAAATCG | *Hind*III | *gr* 5’targeting sequence, R |
|  | 3680^**^ | CGGGGTACCGTTGCTATAAATGCGGGGCGATTATTAGCTG | *Asp718*I | *gr* 3’targeting sequence, F |
|  | 3681^*^ | CCGGATATCCCTTCTTTGATCATATCCCTTATTTTGTC | *EcoR*V | *gr* 3’targeting sequence, R |

*pb* = *P. berghei*, *py* = *P. yoelii*

^*^  Primers used in this study to amplify the complete gr gene

^**^  Primers that have been used to amplify the 5’and 3’UTR target regions of *gr* (Pastrana-Mena *et al*, 2010, J. Biol Chem, 285: 27045-27056)
