# Supplementary material for: A Novel ‘Gene Insertion/Marker Out’ (GIMO) Method for Transgene Expression and Gene Complementation in Rodent Malaria Parasites
Source: PLoS One. 2011 Dec 27;6(12):e29289. doi: 10.1371/journal.pone.0029289 (PMC3246482; doi:10.1371/journal.pone.0029289)
Supplement: Table S2 — Primers used for genotype analysis. (DOC) [file pone.0029289.s005.doc]

**Table S2: Primers used for genotype analysis**

| **No.** | **Primer sequences** | **Restriction sites** | **Description** |
| --- | --- | --- | --- |
| ***Primers for PCR analysis*** | | | |
| 5510 | GCAAAGTGAAGTTCAAATATGTG |  | 5’- intgr *pb230p*, F |
| 5511 | AGTGACTTTCAGTGAAATCGC |  | 3’- intgr *pb230p*, R |
| 3189 | CTGGTGCTTTGAGGGGTG |  | 5’*pbeef1α*, R |
| 4239 | GATTTTTAAAATGTTTATAATATGATTAGC |  | 3’*pbdhfr*, F |
| 4698 | GTTCGCTAAACTGCATCGTC |  | h*dhfr*, F |
| 4699 | GTTTGAGGTAGCAAGTAGACG |  | y*fcu*, R |
| 1637 | AATATGTAGCATTACATTGTCC |  | *pb230p*, F |
| 5600 | ATTCATATCCAACTAAAAAATCTG |  | *pb230p*, R |
| 6527 | GAAGGATATGAATTAGATCCACC |  | 5’- intgr *py230p*, F |
| 6528 | AGACATTGGCATATGAGCAAG |  | 3’- intgr *py230p*, R |
| 4770 | CATCTACAAGCATCGTCGACCTC |  | pL0048, R |
| 4771 | CCTTCAATTTCGGATCCACTAG |  | pL0048, F |
| 6529 | GAGGCCATAGAAAATGATGTAG |  | *py230p*, R |
| 6530 | TTGTTCGAAGTGGGTTCAGG |  | *py230p*, F |
| 4958 | GCATGAACTCCTTGATGATG |  | *mCherry*, R |
| 5515 | GCATGGACGAGCTGTACAAG |  | *mCherry*, F |
| 3173 | TGCCCTTTATTAACTAGTCG |  | 5’*pb*eef1α, F |
| 5514 | CTTGTACAGCTCGTCCATGC |  | *mCherry*, R |
| 6812 | CTCGCAAAGCATTGAACACC |  | *gfp* R |
| 6813 | CTTACCGGAAAACTCGACGC |  | *luciferase*, F |
| 6814 | TGACGGGAACTACAAGACAC |  | *gfp*, F |
| 6815 | ACGAACGTGTACATCGACTG |  | *luciferase*, R |
| 3742 | GGGAAGCTTCGCTAGTTTATATACACGTGG | *Hind*III | *gr* ORF, F |
| 3743 | TCCCCGCGGCATGAACTTTTTCTATTTCTTCTAC | *Ksp*I | *gr* ORF, R |
| ***Primers for PCR probes*** | | | |
| 692 | CTTATATATTTATACCAATTG |  | 3’*pbdhfr/ts,* F |
| 693 | GTTTTTTTTTAATTTTTCAAC |  | 3’*pbdhfr/ts,* R |
| 886 | GGAAGATCTATGGTTGGTTCGCTAAACTGCATCG |  | h*dhfr*, F |
| 887 | GGAAGATCTTTAATCATTCTTCTCATATACTTC |  | h*dhfr*, R |
| 1462 | CATGCCATGGATGAATACTTATTACAGTG |  | *pb25*, F |
| 1463 | CCGGAATTCTTAAATGATATTTGAAAATATTAG |  | *pb25*, R |
| 3680 | CGGGGTACCGTTGCTATAAATGCGGGGCGATTATTAGCTG | *Asp718*I | 3’UTR *gr*, F |
| 3681 | CCGGATATCCCTTCTTTGATCATATCCCTTATTTTGTC | *EcoR*V | 3’UTR *gr*, R |
| ***Primers for qPCR analysis*** | | | |
| 5530 | TTCAGCCTCTGCTTGATCTC |  | *mCherry*, F |
| 6248 | GCGCGTGATGAACTTCGAG |  | *mCherry*, R |
| 6246 | CCAGAATACCCAGGTGTTCTC |  | h*dhfr*, F |
| 6247 | ACATCCGCCAATAGGAACAC |  | y*fcu*, R |
| 6249 | CAATTGCAGGGTTAAATGTTATGAG |  | *pbhsp70*, F |
| 6250 | TTCACCACCTAAATGGGTATCAC |  | *pbhsp70*, R |

*pb* = *P. berghei*, *py* = *P. yoelii;* h = human, y=yeast
